# Effects of Iron Supplements on Heme Scavengers in Pregnancy

**DOI:** 10.4269/ajtmh.20-0413

**Published:** 2021-09-27

**Authors:** Annette M. Nti, Felix Botchway, Hassana Salifu, Juan Carlos Cespedes, Adriana Harbuzariu, John Onyekaba, Christopher Chambliss, Mingli Liu, Andrew Adjei, Pauline Jolly, Jonathan K. Stiles

**Affiliations:** ^1^ Morehouse School of Medicine, Atlanta, Georgia;; ^2^ Korle Bu Teaching Hospital, Accra, Ghana;; ^3^ School of Public Health, University of Alabama at Birmingham, Birmingham, Alabama

## Abstract

In malaria endemic countries, anemia in pregnant women occurs as a result of erythrocyte destruction by *Plasmodium* infections and other causes including malnutrition. Iron supplementation is recommended as treatment of iron-deficiency anemia. Erythrocyte destruction results in increased release of cytotoxic free heme that is scavenged by haptoglobin (Hp), hemopexin (Hx) and heme oxygenase-1 (HO-1). Paradoxically, iron supplementation in pregnant women has been reported to enhance parasitemia and increase levels of free heme. The relationship between free heme, heme scavengers, and birth outcomes has not been investigated, especially in women who are on iron supplementation. We hypothesized that parasite-infected pregnant women on routine iron supplementation have elevated heme and altered expression of heme scavengers. A cross-sectional study was conducted to determine the association between plasma levels of free heme, HO-1, Hp, Hx, and malaria status in pregnant women who received routine iron supplementation and their birth outcomes. Heme was quantified by colorimetric assay and scavenger protein concentration by ELISA. We demonstrated that iron-supplemented women with asymptomatic parasitemia had increased free heme (mean 75.6 µM; interquartile range [IQR] 38.8–96.5) compared with nonmalaria iron-supplemented women (mean 34.9 µM; IQR 17.4–43.8, *P* < 0.0001). Women with preterm delivery had lower levels of Hx (mean 656.0 µg/mL; IQR 410.9–861.3) compared with women with full-term delivery (mean: 860.9 µg/mL; IQR 715.2–1055.8, *P* = 0.0388). Our results indicate that iron supplementation without assessment of circulating levels of free heme and heme scavengers may increase the risk for adverse pregnancy outcomes.

## INTRODUCTION

In 2019, there were an estimated 229 million cases of malaria worldwide and an estimated 409,000 malaria deaths. Infants, children less than 5 years of age, pregnant women, and patients with HIV/AIDS, as well as nonimmune migrants, mobile populations, and travelers are at considerably higher risk of contracting malaria, and developing severe disease, than others.^1^ Clinically, asymptomatic plasmodial infection refers to parasitemia of any density in the absence of fever or other acute symptoms, in individuals who have not recently received treatment. Besides fever, the hallmark of malaria, other symptoms may occur, including nausea, vomiting, or dry cough. Malaria patients can develop severe complications if left untreated, involving central nervous system (cerebral malaria), pulmonary system (respiratory failure), renal system (acute renal failure) and hematopoietic system (severe malaria anemia).^2^^–^^4^ During pregnancy, placental malaria (PM) is characterized by the accumulation of parasitized red blood cells (pRBC) in the placental intervillous space and subsequent prominent infiltration of maternal monocytes/macrophages.^5^ Previous studies in Ghana reported that many pregnant women attending antenatal care were asymptomatically infected with *Plasmodium falciparum*.^6^ The pathogenesis of PM is not completely understood but many studies have associated PM with adverse birth outcomes including high rates of miscarriage, low birth weight (LBW), preterm delivery (PTD), intrauterine growth retardation (IUGR), and neonatal and maternal mortality. A major complication of PM is maternal anemia, which can be attributed to direct destruction of pRBC in symptomatic and asymptomatic individuals.^7^^–^^9^

To address maternal anemia attributed to malnutrition and hemolytic events, pregnant women are routinely prescribed iron to meet increased demands required by pregnancy.^10^^,^^11^ Current WHO guidelines recommend daily iron and folate to reduce anemia and poor pregnancy outcomes.^12^ However, there is controversy about iron supplementation especially in malaria endemic regions.^10^^,^^13^ Reports show that the effects of supplementation in pregnant women are inconsistent and may have negative side effects including gastroenteritis, oxidative stress, and decreased absorption of nonheme iron.^14^^,^^15^ Although reports show both positive and negative maternal and neonatal outcomes, many clinical trials have failed to show clear agreement on the association between iron supplementation and birth outcomes in malaria endemic areas.^11^^,^^16^^,^^17^

These complications may be further exacerbated because of current clinical practice of presumptive iron supplementation without prior screening for ferritin levels in the pregnant women. According to WHO, iron overload is defined as serum ferritin over 150 microgram per liter and iron deficient as under 15 microgram per liter.^18^ Evaluating these levels before iron supplementation may be critical in assessing a pregnant woman’s risk for parasite-associated adverse birth outcomes.

There is a growing body of evidence indicating that low iron stores may protect against plasmodial infection during pregnancy and that iron can paradoxically enhance parasitemia in pregnant women and children. Furthermore, iron supplementation may potentially lead to subsequent adverse birth and perinatal outcomes in women infected with malaria parasites during pregnancy.^19^^–^^21^ Iron is essential to the growth, proliferation, and survival of malaria parasites.^22^ A recent study conducted in the malaria endemic region of Papa New Guinea found that there was a significant reduction of peripheral blood parasitemia in pregnant women who had low peripheral ferritin, and that these women also had reduced risk of LBW babies.^23^ Furthermore, maternal guidelines in the United Kingdom do not recommend iron supplementation for all pregnant women without screening, because of inconsistency in reported benefits and negative side effects of supplementation.^14^

Adding to this body of reported effects of iron supplementation, recent studies indicate that iron supplementation increases circulating free heme in the plasma of Ghanaian pregnant women with asymptomatic plasmodial infection.^24^ Excess free heme is cytotoxic and is a major source of essential iron for parasite growth. The role of free heme in PM as well as its effects on fetal development and birth outcomes remains unclear.

The cytotoxic effects of heme are attenuated by heme scavenger proteins including haptoglobin (Hp), hemopexin (Hx), and heme oxygenase-1 (HO-1). Haptoglobin protects against harmful effects of erythrocyte hemolysis by binding extracellular hemoglobin (Hb).^25^ The Hp–Hb complex is then cleared by CD163 receptors on macrophages. After the exhaustion of Hp, Hx binds extracellular free heme released from degraded free Hb and transports it into circulation. The Hx–heme complex is cleared by receptor-mediated endocytosis via liver parenchymal cells. Previous studies have shown that when plasma levels of Hp and Hx are reduced, endogenous systems against extracellular Hb are overwhelmed. Although HO-1 is the primary enzyme involved in heme degradation and is highly inducible by a variety of stimuli, the most potent inducer of HO-1 is heme.^25^^,^^26^

Heme scavengers are associated with hemolytic anemia and other diseases such as Alzheimer’s and acute kidney injury.^27^^–^^29^ They have been used as biomarkers of cancer progression and therapeutic targets.^30^^,^^31^ However, to date, the potential of heme scavengers as biomarkers of risk associated with malaria in pregnancy or outcomes of the disease remains to be established.

We have previously reported in an in vitro cerebral malaria model that free heme compromises the blood–brain barrier, causing it to be leaky and dysfunctional and responsible for exacerbating cerebral malaria complications.^32^ Using an in vitro model of malaria in pregnancy, we demonstrated that free heme damages trophoblast cells by apoptosis.^33^ Animal studies also show that the heme scavenging system is an essential regulator of placental development.^34^^,^^35^ We have previously reported that iron supplementation elevates circulating free heme levels in the blood of pregnant women in malaria endemic regions and that elevated levels of free heme were associated with adverse birth outcomes.^24^ In this study, we examine the differential expression of heme scavengers in pregnant women based on infection status, iron supplementation, and birth outcomes. We hypothesized that *P. falciparum*–infected pregnant women on routine iron supplementation have elevated circulating heme and altered levels of heme scavengers (Hp, Hx, and HO-1). The relevance of our findings to current protocols for managing plasmodial infection in pregnant women is discussed.

### Variable definitions.

*Uncomplicated pregnancy*: absence of hypertension, preeclampsia, no history of a previous caesarean section and hemorrhage, and a normal presentation of the fetus. *Plasmodium infection*: presence of *Plasmodium* DNA (polymerase chain reaction [PCR]) or antigen (rapid diagnostic testing [RDT]) in the mother’s peripheral blood. *Malaria*: presence of parasites and symptoms of malaria disease. *Anemia*: hemoglobin levels < 11 g/dL of blood. *LBW*: weight < 2,500 g at birth regardless of gestational age. *Full-term delivery (FTD)*: birth between 37 and 42 weeks. *PTD*: delivery occurring before 37 completed weeks of gestation.

## MATERIALS AND METHODS

### Study participants—inclusion and exclusion criteria.

Institutional Review Board (IRB) approval for this study was obtained from Morehouse School of Medicine and the Committee on Human Research, Publications and Ethics from Kwame Nkrumah University of Science and Technology Kumasi, and Korle Bu Teaching Hospital Accra. This cross-sectional study was conducted as part of ongoing National Institutes of Health (NIH)-funded projects on malaria in pregnancy. Subject enrollments took place at multiple sites in Ghana, West Africa. In Accra, three sites were used to enroll participants attending routine antenatal care. These sites included Korle Bu Teaching Hospital, and two satellite clinics including Ussher Polyclinic and Kaneshie Polyclinic. Participants were also enrolled in Kumasi, a centrally located urban city from Komfo Anokye Teaching Clinic. Subjects attending antenatal care were enrolled and provided written consent. After giving consent, participants were given a questionnaire by trained technicians addressing demographic and clinical information. Obstetric and additional clinical information were collected from participants’ hospital records. Iron dosage and regimens were obtained from participant hospital and clinical records. Women included in this study were not assessed for peripheral heme concentration prior to iron supplementation prescription. Women defined in this study as taking iron supplementation, had been placed on regimens by their practitioner, which included 60 mg of iron once a day throughout the duration of their pregnancy, in adherence to current WHO recommendation.^12^ Ferritin levels, as previously described, were not assessed as part of current standard of care and therefore this data were not available for this study. Women who had multiple births, clinical complications, and hemoglobinopathies were excluded from this study.

### Assessment of maternal parasitemia.

Whole blood (8 mL) was collected from participants before delivery for determination of *P. falciparum* antigen test (Histidine-Rich Protein II—Rapid Diagnostic Test [HRPII-RDT]) and to extract DNA. *Plasmodium* DNA was extracted from dried blood spots on filter paper to confirm the presence or absence of malaria parasites. Polymerase chain reaction was used to confirm the presence of *P. falciparum* DNA and exclude *P. vivax*, *P. malariae*, and *P. ovale*. All pregnant women with positive HRPII-RDT and PCR for *P. falciparum* were classified as having plasmodial infection and those with negative HRPII and PCR were classified as plasmodial infection negative.

### Determination of heme and heme scavengers from plasma.

During screening, blood was collected from participants and plasma was separated and stored at −80°C until analysis. To quantify free heme, plasma was centrifuged for 30 minutes at 13,000 rpm to remove contaminating protein and RBCs. Free heme in plasma samples was quantified in *P. falciparum* positive pregnant women and pregnant women with no evidence of parasites using the colorimetric QuantiChrom Heme Assay Kit (Bioassay Systems, Hayward, CA) according to manufacturer protocol. Free Hp and free Hx were quantified in plasma samples with Human Haptoglobin ELISA Kit and Human Hemopexin ELISA Kit, respectively (Genway Biotech Inc, San Diego, CA), whereas HO-1 was quantified with HO-1 Human ELISA kit (Enzo Life Sciences, Farmingdale, NY). Data were then analyzed by Spectra Max 190 fluorescence micro plate reader at 450 nm wavelength. Birth outcome information was obtained after delivery by cell phone follow-up and subsequently validated with participants’ clinical records. Demographic, clinical, and obstetric data were used for correlation analyses. We assessed specificity and sensitivity for heme, HO-1, Hp, and Hx as potential biomarkers of heme scavenging capability. Ratios of heme and heme scavenger biomarker concentrations were calculated as additional means to discriminate between pregnant women with or without plasmodial infections.

### Statistical analysis.

Student’s *t*-test was used for all normal continuous data comparisons. Mann–Whitney was used for nonparametric continuous data analysis. Categorical data were compared by Pearson χ^2^ analysis. Receiver operator characteristic (ROC) curves were used to assess specificity and sensitivity of heme and heme scavenger biomarkers in predicting birth outcomes. All analyses were performed with SAS 7.1 software and GraphPad Prism 8. Analysis with *P* value of < 0.05 was considered statistically significant.

## RESULTS

### Demographic, obstetric, and clinical characteristics based on RDT outcome.

A total of 145 women were included in this study with a median age of 27 years. Out of these women, 101 were negative for the HRPII-RDT and 44 were positive for the HRPII-RDT. Since the 44 women had no malaria symptoms and were not receiving antimalaria treatment at the time of blood draw they were classified as asymptomatic. Majority of women with confirmed plasmodial infection had no more than a junior high school education. In terms of income, among women with asymptomatic plasmodial infection, a greater percentage of women earned less than $25 a week compared with women who did not have plasmodial infection. Majority of participants who had plasmodial infection were nulliparous. More pregnant women with asymptomatic infection took herbal supplements, vitamins, folic acid (77.3%, *P* < 0.0001) and iron supplementation (70.4%, *P* < 0.0001) compared with noninfected women (Table 1).

**Table 1:** Description of pregnant women in this study who were RDT negative (Neg) or RDT positive (Pos)

Demographic, obstetric, clinical characteristic based on RDT outcome			
Variable	RDT Neg *N* = 101 (%)	RDT Pos *N* = 44 (%)	*P* value
Age	27.8 (18–42)	27.64 (17–39)	0.86
Marital status			
Single	32 (31.6)	18 (40.9)	0.076
Married	69 (68.3)	26 (59.0)	
Education			
Junior HS or less	61 (60.3)	32 (72.7)	0.095
High school or more	40 (39.6)	12 (27.2)	
Income (USD/week)			
< 25	70 (70.4)	31 (30.6)	0.89
> 25	31 (30.6)	13 (13.4)	
Parity			
Nulliparous	26 (25.7)	10 (22.7)	0.57
Primiparous	26 (25.7)	8 (18.2)	
Multiparous	50 (49.5)	7 (15.9)	
Taking vitamins/FA	37 (36.6)	34 (77.3)	< 0.0001
Not taking vitamins FA	64 (63.3)	10 (22.7)	
Iron supplementation	30 (29.7)	31 (70.4)	< 0.0001
No iron supplementation	70 (69.3)	13 (29.5)	

FA = folic acid; RDT = rapid diagnostic testing. Mean and group percentage (%) for each variable are given. Pearson χ^2^ was used for statistical comparison among women. Statistical significance was set at *P* < 0.05.

### Hematological characteristics of participants based on malaria status.

Complete blood counts (CBC; Table 2) indicated that majority of the women who were RDT positive were anemic. Mean corpuscular volume (MCV) was significantly lower for women who were RDT negative compared with women who tested RDT positive. Mean corpuscular hemoglobin concentration (MCHC) was also significantly lower in RDT-positive women. Platelet count was significantly lower in RDT-positive women when compared with women who were RDT negative, in accord with previous studies.^6^^,^^24^

**Table 2:** Hematological description of pregnant women in this study

Hematological characteristics of participants based on malaria status			
	Non malaria (*N* = 101)	Malaria (*N* = 44)	*P* value
Demographic			
Age	27.8(18–42)	27.6 (17–39)	0.86
Clinical			
Anemic (Hgb < 11 g/dL)	38 (37.623%)	20 (45.45%)	0.47
WBC (10_3_/μL)	7.4 ± 0.63	7.4 ± 0.48	0.99
RBC (10_6_/μL)	4.4 ± 0.07	4.1 ± 0.13	0.51
Hemoglobin (g/dL)	11.4 ± 0.32	11.2 ± 0.20	0.24
Hematocrit (%)	36.0 ± 1.06	34.8 ± 0.52	0.26
MCV (fL)	89.5 ± 1.56	84.1 ± 0.82	< 0.001
MCH (pg)	28.4 ± 0.60	27.9 ± 0.54	0.58
MCHC (g/dL)	32.9 ± 0.48	31.8 ± 0.55	0.02
Platelet (10_3_/μL)	212 ± 6.59	184.8 ± 7.14	< 0.001

MCH = mean corpuscular hemoglobin; MCHC = mean corpuscular hemoglobin concentration; MCV = mean corpuscular volume; RBC = red blood cell; WBC = white blood cell. Pearson χ^2^ was used for statistical comparison among women. Clinical mean and SD results reported. Statistical significance was set at *P* < 0.05.

### Circulating heme and heme scavenger levels in pregnant women with asymptomatic plasmodial infection.

**Figure 1.**
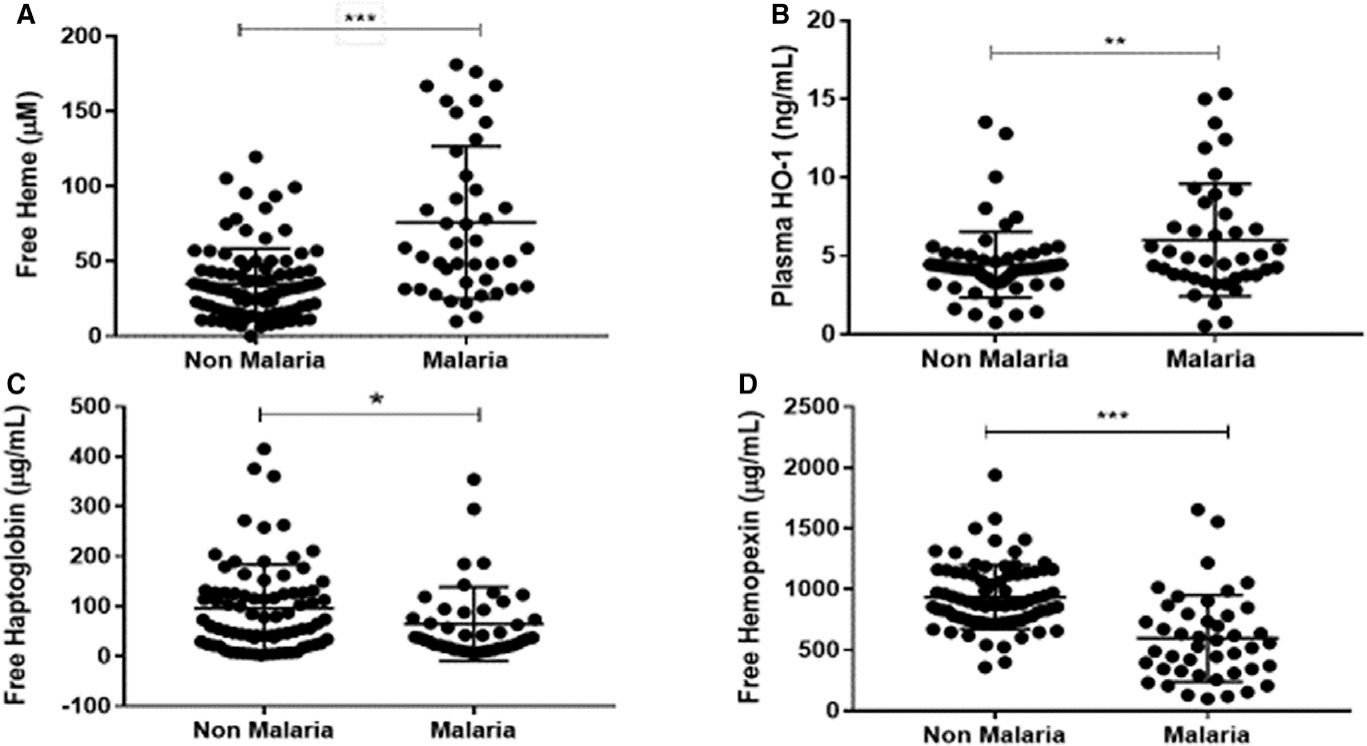
Heme and heme scavenger plasma concentrations in rapid diagnostic testing (RDT)-positive and RDT-negative pregnant women. Plasma concentration levels among pregnant women who tested RDT positive and were asymptomatic, and women who had a negative RDT test. (**A**) Free plasma heme, (**B**) free plasma heme oxygenase-1 (HO-1), (**C**) free plasma Haptoglobin, and (**D**) free plasma Hemopexin. Unpaired *t* test was used to compare for statistical differences in levels among pregnant women. Statistical significance was set at *P < 0.05, **P < 0.01, ***P < 0.001.

As expected, pregnant women with plasmodial infection (RDT-positive) had increased mean plasma concentration of free heme (75.76 µM ± 7.833) compared with RDT-negative women (34.88 µM ± 2.372). Mean plasma concentration of HO-1 in RDT-positive women (6.014 ng/mL ± 0.554) was significantly higher than RDT-negative women (4.441 ng/mL ± 0.253). Rapid diagnostic testing (RDT)-positive women had significantly lower levels of Hp (64.35 µg/mL ± 10.99) and Hx (593.9 µg/mL ± 53.71) than RDT-negative women (Hp 95.53 µg/mL ± 9.789; Hx 932.6 µg/mL ± 28.96) (Figure 1).

### Demographic, obstetric, and clinical characteristics by term delivery.

In terms of PTD, there were significant differences in age with younger women giving birth before 37 weeks gestational age as illustrated in Table 3. Women who did not carry to full term because of spontaneous abortion or miscarriage were excluded from the analysis. There were more married women with less than a high school education making less than $25 per week that were more likely to have PTD compared with those making more than $25 per week. A greater percentage of women with plasmodial infection were more likely to have PTD (*P* = 0.0038). Moreover, 60% of women with PTD were taking iron supplements in comparison to 47% of women who had FTD (not statistically significant).

**Table 3:** Description of pregnant women in this study who had a full-term delivery or preterm delivery

Demographic, obstetric, clinical characteristics by term delivery			
	Full-term delivery (%)	Preterm delivery (%)	*P* value
Age	29 (17–41)	24 (18–38)	0.01
Marital status			
Single	18 (23.68)	9 (45.20)	0.08
Married	55 (72.36)	11 (55.80)	
Education			
Junior high school or less	49 (64.47)	14 (73.68)	0.45
High school or more	26 (35.53)	6 (26.32)	
Income (dollars/week)			
< 25	52 (71.3)	17 (77.2)	0.58
> 25	21 (28.7)	5 (22.8)	
Parity			
Nulliparous	15 (21.74)	9 (45.2)	0.36
Primiparous	17 (24.64)	6 (30.8)	
Multiparous	37 (53.62)	5 (25.0)	
Plasmodial infection	25 (32.89)	10 (50.0)	0.004

Pearson χ^2^ was used for statistical comparison among women. Statistical significance was set at *P* < 0.05.

### Circulating levels of free heme and heme scavengers correlate with birth outcomes.

**Figure 2.**
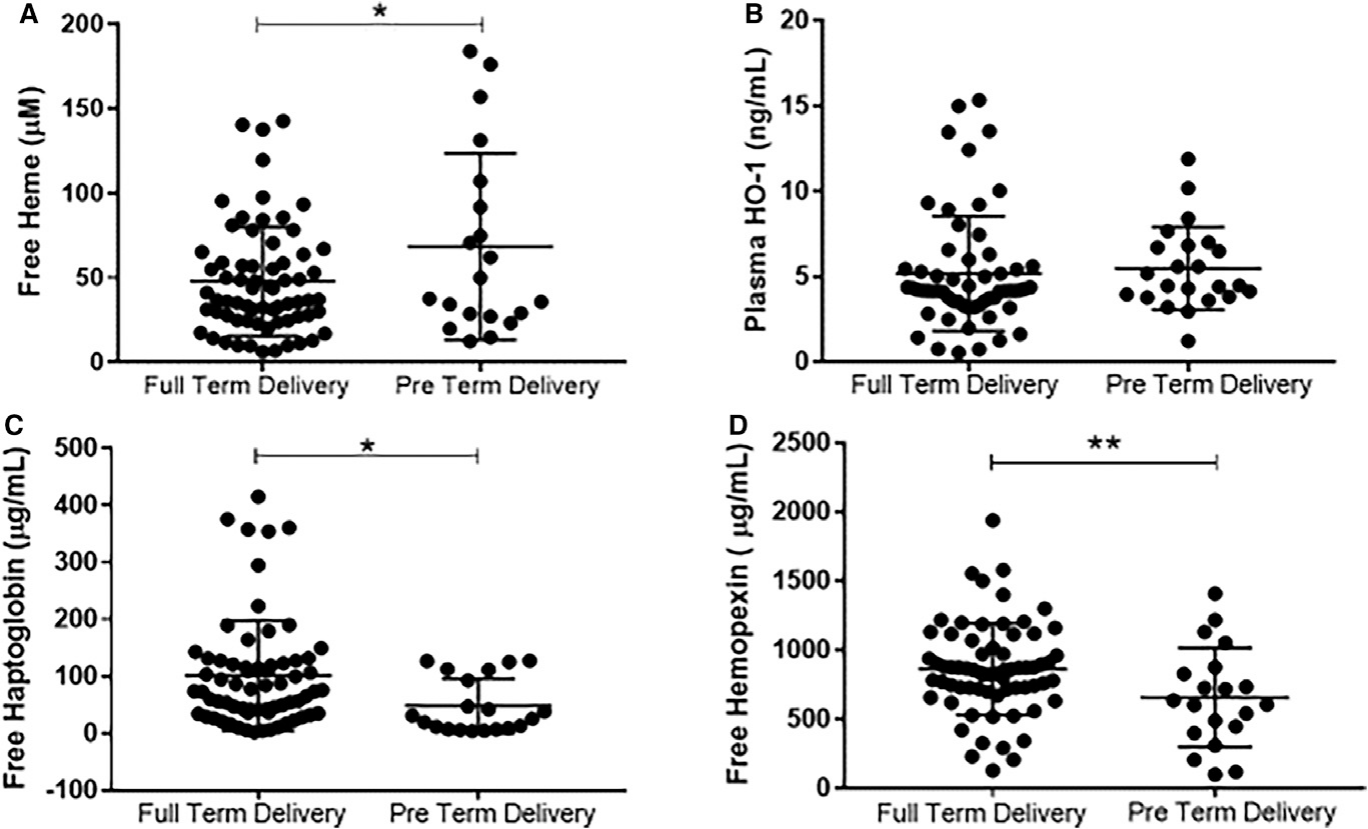
Heme and heme scavenger plasma concentrations in women who had full-term delivery compared with women with preterm delivery. Plasma concentration levels among women with full-term delivery and women who had preterm delivery (**A**) Free plasma heme, (**B**) free plasma heme oxygenase-1 (HO-1), (**C**) free plasma Haptoglobin, and (**D**) free plasma Hemopexin. Unpaired *t* test was used to compare for statistical differences in levels among pregnant women. Statistical significance was set at *P < 0.05, **P < 0.01, ***P < 0.001.

Mean plasma-free heme (68.4 ± 12.3 µM) for women who had PTD was elevated compared with women with FTD (47.8 ± 3.9 µM). Mean plasma levels of free HO-1 however were not significantly elevated in women who had PTD (5.5 ± 0.51 ng/mL) compared with those who had a FTD (5.2 ± 0.44 ng/mL). However, mean plasma levels of free scavengers (Hp and Hx) decreased in women with PTD compared with women who had FTD. Mean plasma-free Hp of women who had PTDs (48.2 ± 10.7 µg/mL) was lower than women with FTDs (101.5 ± 11.8 µg/mL). Similarly, mean plasma-free Hx levels for women who had PTDs (656.0 ± 80 µg/mL) was also lower than those with FTDs (860.9 ± 40.2 µg/mL) (Figure 2).

### Ratios of heme and heme scavengers by plasmodial infection and birth outcomes.

To assess the relationships between the heme and heme scavenger ratios and plasmodial infection and birth outcomes, we analyzed the ratios of participant heme and scavenger plasma concentration levels. The results in Table 4, show that ratios for heme to respective scavengers HO-1, Hp, and Hx were all significantly different between RDT-negative and RDT-positive women. Heme:HO-1 ratio (*P* = 0.004) increased. However, Heme:Hp (*P* = 0.014) and Heme:Hx (*P* = 0.012) ratios were significantly decreased in RDT-positive women. Also, the HO-1:Hx ratio (*P* = 0.0007) significantly decreased in RDT-positive women in comparison to RDT-negative women.

**Table 4:** Comparison of heme and plasma scavenger median ratios between women with or without plasmodial infection

Ratios of heme and heme scavengers by plasmodial infection			
	No plasmodial infection	Plasmodial infection	*P* value
Heme:HO-1	7.71	11.70	0.004
Heme:Hp	2.30	0.66	0.01
Heme:Hx	32.40	8.19	0.01
HO-1:Hp	17.51	7.92	0.18
HO1-Hx	219.70	100.60	0.0007
Hp:Hx	15.92	14.09	0.79

HO-1 = heme oxygenase-1; Hp = haptoglobin; Hx = hemopexin. Statistical significance was set at *P* < 0.05.

Heme and heme scavenger ratios were also assessed with respect to gestational age at the time of delivery in Table 5. The HO-1:Hx ratio (*P* = 0.003) was significantly decreased in women who had PTD in comparison to women who had FTD.

**Table 5:** Comparison of heme and plasma scavenger ratios between women who had full-term deliveries and women who had preterm deliveries

Ratio of heme and heme scavengers by term delivery			
	FTD	PTD	*P* value
Heme:HO-1	9.22	10.69	0.60
Heme:Hp	1.18	1.97	0.85
Heme:Hx	22.67	12.51	0.06
HO-1:Hp	17.09	8.02	0.16
HO1-Hx	202.6	124.10	0.003
Hp:Hx	13.16	26.96	0.35

FTD = full-term delivery; HO-1 = heme oxygenase-1; Hp = haptoglobin; Hx = hemopexin; PTD = preterm delivery. Statistical significance was set at *P* < 0.05.

### Heme and heme scavenger ratios by iron supplementation status.

**Figure 3.**
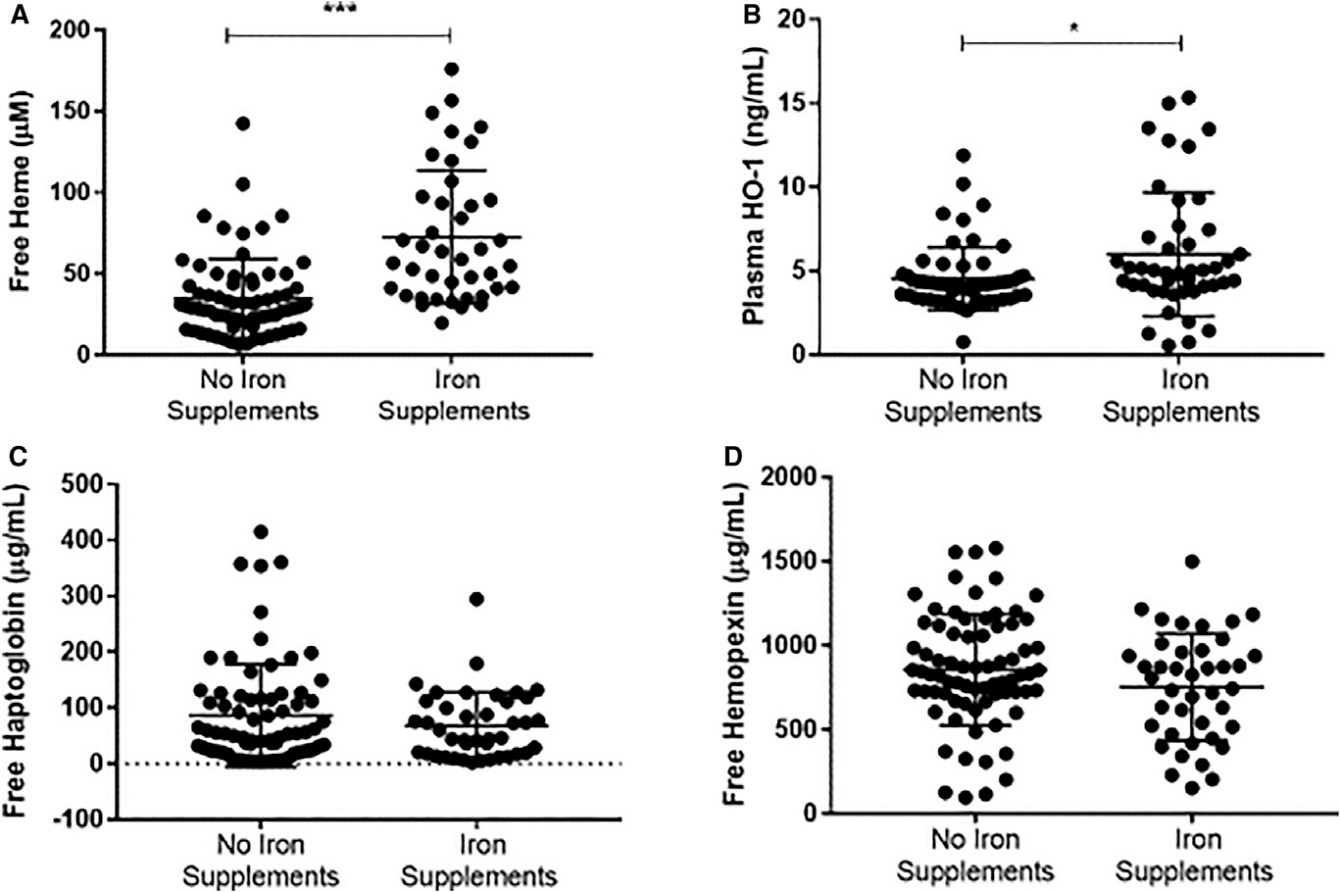
Heme and heme scavenger plasma concentrations in women taking iron supplementation and women who were not taking iron supplementation. Plasma concentration levels among women who were taking iron supplementation during pregnancy and women who were not taking iron supplementation (**A**) Free plasma heme (**B**) free plasma heme oxygenase-1 (HO-1), (**C**) free plasma Haptoglobin, and (**D**) free plasma Hemopexin. Unpaired *t* test was used to compare for statistical differences in levels among pregnant women. Statistical significance was set at *P < 0.05, **P < 0.01, ***P < 0.001.

We measured the levels of heme and heme scavengers in women who reported intake of iron supplementation and found that iron supplementation increased the levels of free heme in plasma (72.6 ± 6.4 µM) when compared with women who reported no intake of iron supplementation (34.9 ± 2.9 µM). However, mean plasma levels of HO-1 were significantly increased in women who were taking iron supplementation (67.8 ± 9.3 ng/mL) compared with women who were not (4.5 ± 0.25 ng/mL). Mean plasma levels of Hp were not significantly decreased in women taking iron supplementation (67.8 ± 9.3 µg/mL) compared with those who were not (86.1 ± 10.7 µg/mL). Similar results were found with mean plasma levels of Hx with no significant decrease in women taking iron supplementation (752.8 ± 49.4 µg/mL) compared with women not taking iron supplementation (855.9 ± 38.3 µg/mL) (Figure 3).

After assessing the ratios of heme and corresponding scavengers in terms of iron supplementation, we determined that there was a significant increase in heme:HO-1 ratio (*P* = 0.05), a significant decrease in heme:Hx ratio (*P* = 0.01). Additionally, the results of this analysis found a significant decrease in the HO-1:Hx ratio (*P* = 0.008) between women on iron supplements and women who were not on iron supplements, as shown in Table 6.

**Table 6:** Comparison of selected heme and respective plasma scavenger median ratios between those who were taking iron supplementation and those who were not

Heme and heme scavengers ratios by iron supplementation status			
	No iron	Iron	*P* value
Heme:HO-1	7.14	11.66	0.05
Heme:Hp	1.95	0.79	0.06
Heme:Hx	31.69	12.15	0.01
HO-1:Hp	14.18	9.72	0.52
HO1-Hx	215.70	140.10	0.009
Hp:Hx	18.69	18.03	0.94

HO-1 = heme oxygenase-1; Hp = haptoglobin; Hx = hemopexin. Statistical significance was set at *P* < 0.05.

### Specificity and sensitivity of heme and heme scavengers as biomarkers for asymptomatic infection and PTD.

**Figure 4.**
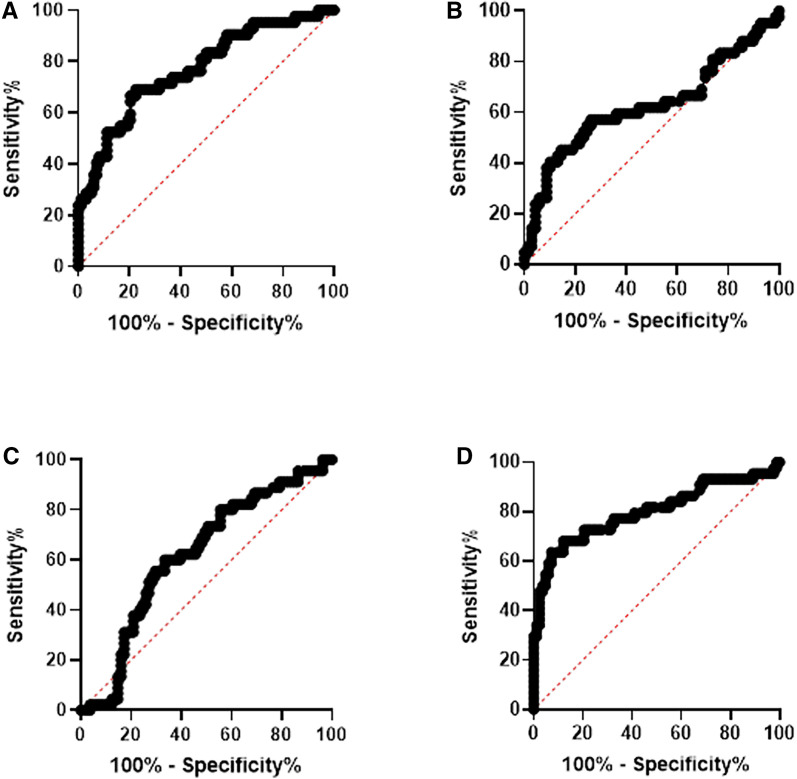
Receiver operator characteristic (ROC) curves for heme, heme scavengers, and malaria. The ROC curve for (**A**) heme, (**B**) heme oxygenase-1 (HO-1), (**C**) Haptoglobin, and (**D**) Hemopexin and malaria. Statistical significance was set at *P* < 0.05. This figure appears in color at www.ajtmh.org.

Receiver operating characteristic curves were constructed to assess the sensitivity and specificity of using heme, Hp, Hx, and HO-1 as biomarkers of asymptomatic infection and PTD in pregnant women who took iron supplementation. Figure 4 illustrates the association of heme, HO-1, Hp, and Hx, respectively, with asymptomatic infection. Heme: AUC 0.80 *P* < 0.0001, HO-1: AUC 0.63 *P* = 0.028, Hp: AUC 0.62 *P* = 0.027, and Hx: AUC 0.81 *P* < 0.001 (Figure 4).

**Figure 5.**
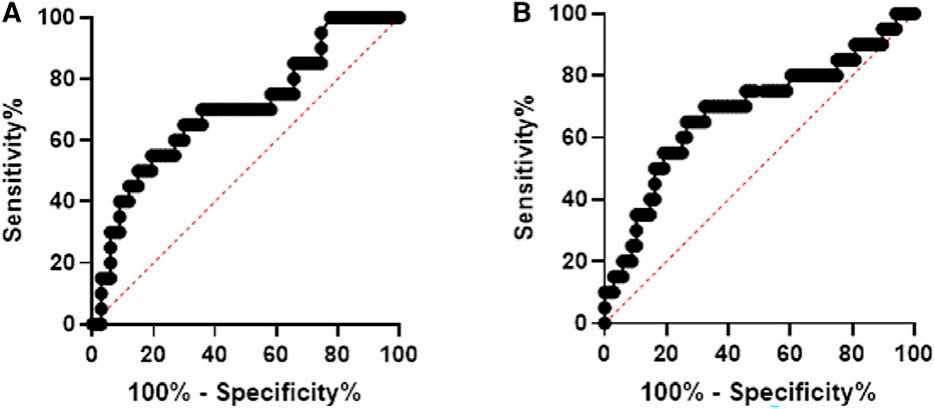
Receiver operator characteristic (ROC) curves for heme scavengers and preterm delivery (PTD). The ROC curve for (**A**) Haptoglobin and (**B**) Hemopexin and PTD. Statistical significance was set at *P* < 0.05. This figure appears in color at www.ajtmh.org.

Figure 5 illustrates the association of heme and heme scavenger levels with PTD. Although assessments were done for heme and HO-1, the results are shown for Hx and Hp: Hp AUC 0.7207 *P* = 0.001 and Hx AUC 0.7001 *P* = 0.013 (Figure 5).

**Figure 6.**
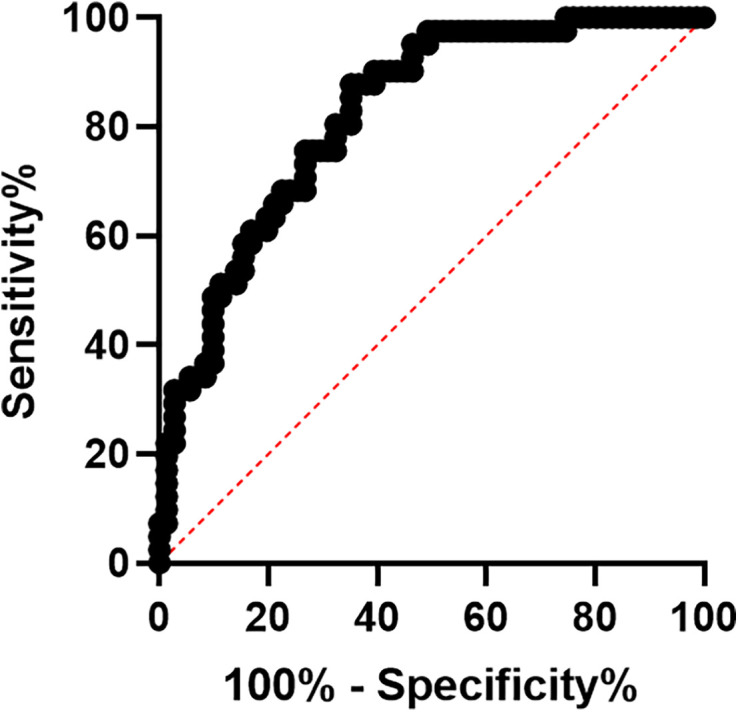
Receiver operator characteristic (ROC) curve for heme and iron supplementation. The ROC curve for heme and iron supplementation. Statistical significance was set at *P* < 0.05. This figure appears in color at www.ajtmh.org.

Although ROC curve assessments were done for all parameters previously described, Figure 6 shows the heme curve for women with asymptomatic infection who were taking iron supplementation (Heme: AUC 0.82 *P* < 0.0001) (Figure 6).

## DISCUSSION

There is much evidence that plasmodial infection during pregnancy is associated with adverse birth outcomes such as LBW and PTD. We have previously showed that women with increased plasma-free heme were more likely to have PTD. It was also demonstrated that pregnant women who took iron supplements had higher than basal levels of heme and HO-1.^24^ This led us to hypothesize that women with adverse pregnancy outcomes have high free plasma heme that alters heme scavenger levels as well as heme scavenging capacity. The results of this current study has shown that circulating free heme levels are elevated not only in pregnant women with symptomatic *P. falciparum* infections, but also in pregnant women who are asymptomatic, take iron supplementation and have PTD.^24^ Circulating heme:HO-1, heme:Hp, heme:Hx, and HO-1:Hx ratios differentiated between women who were noninfected and those with asymptomatic infection. HO-1:Hx ratios remained significant for PTD, which makes this ratio a potential indicator (biomarker) of efficient heme scavenging in pregnant women who take iron supplementation.

We examined the importance of heme-scavenging molecules in this study because of their remarkable role in attenuating deleterious effects of heme in the pathogenesis of malaria. The pathogenesis of malaria is not exclusively because of parasitemia but also involves parasite and host-derived or parasite-induced factors such as heme, HO-1, Hp, and Hx. Heme is a by-product of hemolysis or myolysis that can occur as a result of various pathological states including sickle disease, ischemia reperfusion, stroke, or malaria.^26^ Although heme is an essential molecule to aerobic organisms and is essential to many biological reactions, excess free heme can lead to damage as a result of deleterious toxicity to cells, tissues, and organs.^36^ Heme causes systemic damage by contributing redox-active iron that is involved in the Fenton reaction and produces toxic-free hydroxyl radicals that ultimately damage lipid membranes, proteins, and nucleic acids.^37^ This eventually activates cell signaling pathways, pro-inflammatory transcription factors, changes protein expression, and perturbs membrane channels.^26^^,^^38^

The effects of elevated circulating free heme are countered by heme scavengers including HO-1, Hp, and Hx. Heme oxygenase-1 is a heme catabolizing enzyme that converts heme into iron, biliverdin, and carbon monoxide (CO).^39^^,^^40^ Haptoglobin forms a complex with free hemoglobin that is released during a hemolytic event and transports the captured molecule to macrophages where it is bound to scavenger CD163.^36^ When this scavenging mechanism is overwhelmed, free hemoglobin is rapidly oxidized to methemoglobin that subsequently releases free heme.^41^ To address this increase in free heme, Hx maintains lipophilic heme in a soluble state that is essential to the reutilization of heme-bound iron.^42^ Hemopexin is an acute phase protein that responds in activation to trauma, infection, stress, or inflammation.^43^ Binding of free heme is essential to limiting the amount of heme available to catalyze radical formation and also decreases available essential iron necessary for parasite multiplication.^44^ Although not assessed in this study, albumin also complexes with heme to aid in the avoidance of the toxic effects of free heme in blood. When Hx is exhausted, heme wholly binds to albumin.^45^ Overexpression of HO-1 is associated with the resolution of inflammation by heme degradation that results in CO, bilirubin, and ferritin.^34^ Bilirubin is an efficient scavenger of peroxyl radicals that ultimately inhibits lipid peroxidation and attenuated oxidative stress and ultimately cell death.^46^ Carbon monoxide mediates anti-inflammatory effects by inhibiting expression of pro-inflammatory cytokines and prevents vaso-occlusion and subsequent vascular inflammation.^26^

Therefore, the scavenging capacity of these three proteins is important, especially in individuals with ongoing erythrocyte hemolysis because of plasmodial infection. Any dysfunction in this heme-scavenging cascade in plasmodial infected pregnant women, for example, may limit effective heme clearance capacity and give way to heme damage to the placental barrier. At a cellular level, a study done by Liu et al indicated that treatment of placental trophoblast cells with exogenous heme–induced apoptosis and inhibited cell fusion through activation of the STAT3 pathway.^33^ If indeed parasite-induced increases in circulating heme damages trophoblast cells to comprise the placental barrier components in vivo as observed in vitro, then it will be vital that pregnant women possess efficient scavenging mechanisms to effectively clear free heme to avoid injury to the placenta.^33^

We previously reported that pregnant women with asymptomatic plasmodial infection had higher plasma levels of free heme and HO-1 than uninfected pregnant women. This study found an association between women taking iron supplementation and higher levels of heme and HO-1.^24^ Our results confirmed these findings and also showed that although there are higher levels of heme and HO-1, there was no significant difference in Hp and Hx between pregnant women who did not take iron and pregnant women who did take iron supplements.

A closer evaluation of the relationship between HO-1 and Hp reveals that HO-1 is released in a dose-responsive manner in response to Hp release. In women who had PTD, there was significantly lower ratio of HO-1:Hx, which may indicate that these women may not have adequate Hx to stimulate release of HO-1 to bind heme to Hx or for conversion to a less deleterious form by HO-1. Because there is evidence that heme causes apoptosis and damage to trophoblast cells that make up the placental barrier, this may point to inflammation and damage that could possibly lead to PTD.

Although there are many molecular pathways that lead to PTD, it has been recognized that maternal infections, such as *P. falciparum*, increases the risk of preterm birth among pregnant women.^47^ More recently, there is a growing body of evidence suggesting that intrauterine infection as well as inflammation are commonly associated with preterm birth.^48^ It has been demonstrated that heme activates apoptosis and pro-inflammatory factors through the TLR4 pathway which is expressed in the uterus.^26^^,^^49^ An increase in several pro-inflammatory cytokines and mediators have been linked to PTD and stimulating premature uterine contractility.^48^

However, the deleterious effects of free heme are modulated by heme scavengers, and a recent study illustrated that HO-1 protein and activity were increased with infusion of Hp and Hx. That study used a hyper hemolytic sickle cell disease (SCD) mouse model to examine cellular response to Hp and Hx supplementation.^50^ In addition, they also showed that HO-1 is increased in a dose-dependent manner in response to increased Hp and Hx infusion. Furthermore another study concluded that Hx induced the expression of HO-1 and protected blood–brain barrier integrity in an ischemic rat model.^51^ These findings may be indicative of the significance of differentially expressed HO-1:Hx ratios found in this study. There is a lack of predictive PM biomarkers for at-risk individuals. The differential expression of Hx:HO-1 may provide a basis to assess pregnant women who may be at risk of inefficiently scavenging heme and therefore may be more susceptible to lower expression of HO-1 and would not benefit from the protective and anti-inflammatory actions of HO-1.

It is also important to note that individuals with asymptomatic plasmodial infection may have a high risk of developing symptomatic malaria although it is suggested that asymptomatic parasitemia may confer partial immunity against more severe forms of malaria.^6^^,^^52^ Also, many asymptomatic cases do not receive treatment, which may eventually lead to adverse consequences of symptomatic malaria such as the chronic and extensive destruction of RBCs.^53^ Iron supplementation in asymptomatic women may further exacerbate this condition by providing high levels of iron that promote parasite replication.^13^ Current clinical practice does not screen for serum ferritin before iron supplementation. This point is critical in that some pregnant women may already have sufficient iron, and further supplementation may lead to excessive iron in circulation, which could potentially provide an iron reservoir for parasites. These women are also not screened for heme or heme scavengers before iron supplementation. As we have shown in this study, women on iron supplementation had an increase in free heme. We have detailed the consequences of excess free heme, but we emphasize how a lack of screening of ferritin and heme can lead to excess free heme and subsequent adverse outcomes such as PTD.

Investigating host factors that mediate disease pathogenesis is critical not just for malaria but also other hemolytic diseases and hemoglobinopathies. Current treatment strategies address parasite-derived factors but may not account for the contribution of detrimental host factors such as heme. Current therapy to reduce malaria morbidity and mortality in pregnancy recommends three doses of intermittent preventative therapy with sulfadoxine pyrimethamine starting as early as the second trimester.^54^ However, aside from compliance issues, such approaches in treatment addresses the reduction of parasite burden but does not address heme burden, or inefficiencies in host scavenging mechanisms, which may play a critical role on outcomes.

In the future, we will investigate the role of variations such as single nucleotide polymorphisms (SNPs) in genes involved in the heme scavenging system and their impact on pregnancy outcomes. In this study, we have determined that lower ratios in HO-1:Hx are associated with asymptomatic plasmodial infections and PTD. Women who have SNP(s) in genes encoding for proteins involved with heme scavenging that limit their capacity to efficiently scavenge heme may potentially manifest adverse maternal and birth outcomes.

This study is part of a larger overall assessment of the role of free heme in adverse pregnancy outcomes and was not designed as an iron supplementation clinical trial. Thus, at the time of submission of the manuscript, the investigators did not have information on ferritin levels. This remains a limitation of this study. The chronicity and episode occurrence of plasmodial infection was also not known for participants at the time of publication of this data. These factors presented limitations to the analyses and conclusions of this study. Nevertheless, the results indicate a strong rationale for assessing plasmodial infection even in asymptomatic pregnant women before iron supplementation. The comparative ratios assessed in this study provide a foundation for developing surrogate diagnostic tools to assess biomarkers that mark the efficiency or lack thereof of heme scavengers in asymptomatic pregnant women. Measuring ratios in the plasma of pregnant women is a minimally invasive option that will not only provide concentrations of these molecules, but also assist in drawing possible conclusions on the risks and benefits of iron supplementation and the risk of adverse outcomes on a personalized basis in asymptomatic plasmodial infections in pregnant women.
